# Supplementary material for: Integrating epigenomic data and 3D genomic structure with a new measure of chromatin assortativity
Source: Genome Biol. 2016 Jul 8;17:152. doi: 10.1186/s13059-016-1003-3 (PMC4939006; doi:10.1186/s13059-016-1003-3)
Supplement: Additional file 1: — A PDF file containing all supplementary texts, figures and tables. (PDF 4177 kb) [file 13059_2016_1003_MOESM1_ESM.pdf]

Additional file 1

Chromatin assortativity: Integrating epigenomic data and 3D genomic structure

Vera Pancaldi[1], Enrique Carrillo de Santa Pau[1], Biola Maria Javierre[2], David Juan[1], Peter Fraser[2], Mikhail Spivakov[2], Alfonso Valencia[1], Daniel Rico[1]

[1]Spanish National Cancer Research Centre (CNIO), Madrid, Spain; [2]The Babraham Institute, Cambridge, United Kingdom

Table of Contents

Figure S1: PCHi-C chromatin Interaction Network....2
Text S1. Robustness and significance of ChAs values....3
Figure S2: ChAs robustness to edge and node removal....5
Figure S3: Schematic description of randomization procedures....6
Figure S4: Results of randomization procedures....7
Figure S5: ChAs correlation of network after distance filtering....8
Text S2 Comparison of ChAs with traditional approaches....9
Figure S6: Comparison of ChAs with enrichment of features in important nodes....10
Text S3: Network community finding....11
Figure S7: Enrichment of features in chromatin communities....12
Figure S8: HiCap chromatin interaction network....13
Figure S9: ChIA-PET networks, SMC1 and RNAPII and network statistics....14
Figure S10: Comparison of ChAs in 3 networks against PCHiC....16
Figure S11: Comparison of ChAs in 3 networks against HiCap and RNAPII ChIA-PET vs SMC1 ChIA-PET....17
Figure S12: Comparison of ChAs P-P vs. P-O....18
Figure S13 Core and periphery....20
Text S4: Party and date hubs....21
Figure S14 Party/date hubs....22
S15 Heatmap of correlations between enrichment of features in different chromatin communities. 23
Figure S16: ChAs and abundance for different RNAPII variants in P-P and P-O subnetworks and their rewirings....24
Figure S17: Abundance of features in Other end fragments with no H3K4me1 enhancer mark....26
Figure S18: Comparison of presence of different RNAPII variants in P and O fragments....27
Table S1: Overlap of fragments in different chromatin interaction networks....28

Figure S1: PCHi-C chromatin Interaction Network

Entire PCHi-C Network obtained with ChiCAGO.

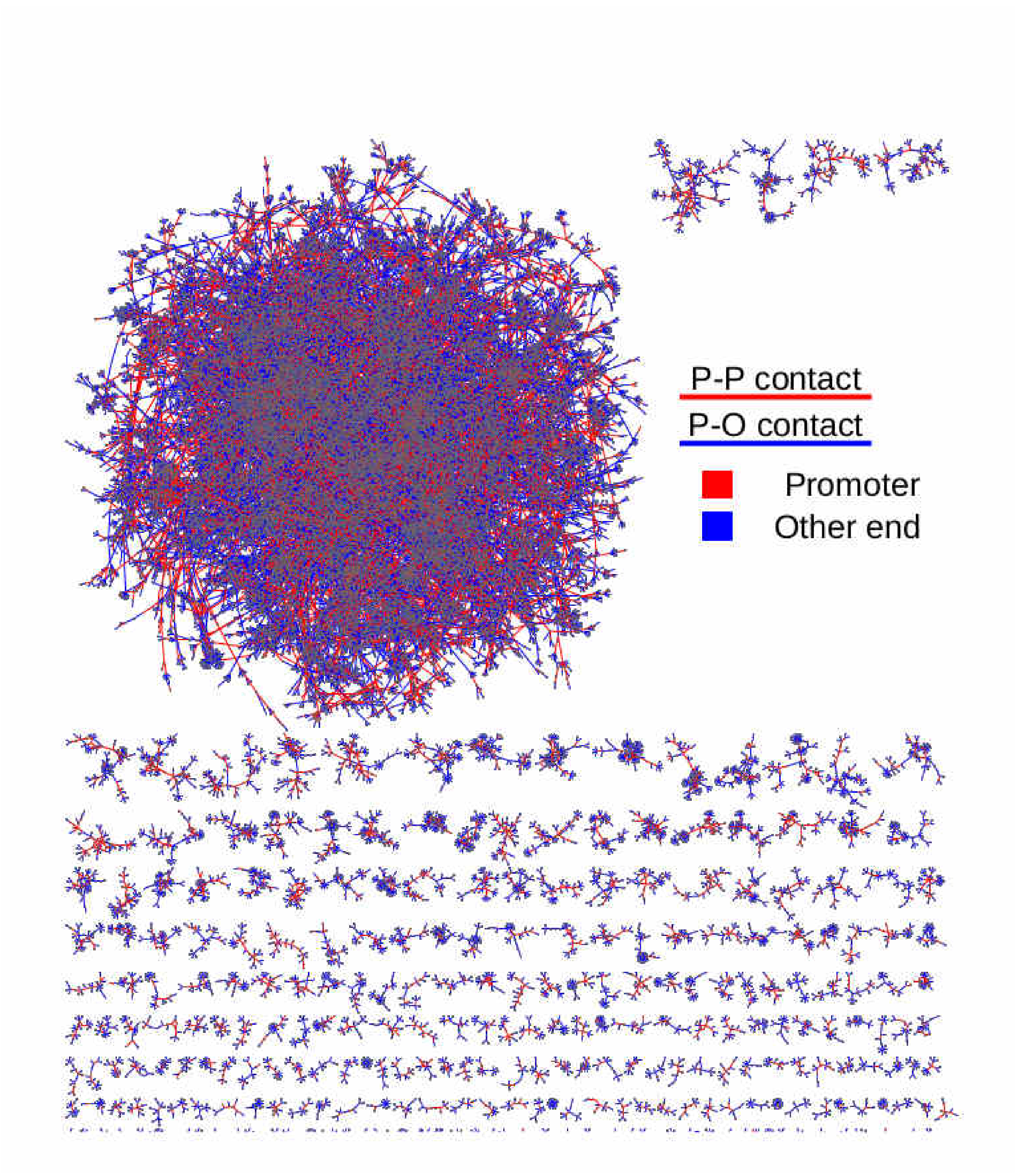

Text S1. Robustness and significance of ChAs values

We assessed how the ChAs values can be affected by the accuracy of the topology of the chromatin interaction network (**Figure S2**). To this end we performed random removal of edges from the PCHi-C network and observed very stable values of ChAs for all features, even when removing 70% of the edges (**Figure S2A**). On the contrary, removing preferentially nodes with high abundance of the EZH2 feature (a member of PcG) produced an almost complete loss off assortativity in this mark (bringing it below values we consider significant, see below) after removing only 10% of the total network edges (**Figure S2D**). As could be expected, removing these nodes also affects the ChAs of other related features (other components of PcG) which show a similar decline.

To judge whether the ChAs values obtained in a specific network are significantly different from random, we perform random permutations of the assignment of features to the fragments (see schematic in **Figure S3**) and check whether the observed ChAs is different from the randomized version. We perform 100 permutations and calculate in how many of these permutations we observe a ChAs higher than what is observed in our original datasets, thus calculating an empirical p-value. This randomization preserves the overall abundance of each feature as well as the topology of the network (see schematic description in **Figure S3, panel II**) and shows that the values of ChAs obtained for the PCHi-C network are significantly higher than those expected at random for 69 out 78 features (**Figure S4A, Additional File4**).

We also performed an alternative type of randomization on the subnetworks inspired by the approach used in Sahlen et al. (Sahlén et al., 2015), in which we preserve the chromosomal feature correlation (see schematic description in **Figure S3, panel III**). Referring to chromosome 1, we first bin chromosomal distances spanned by each interaction into 20 intervals. Then for each promoter-other end interaction we pick a fictitious other end in the same distance bin, but randomizing the start coordinate and keeping the original length of the fragment, thus creating a new interaction. We repeat this 50 times and again estimate how often the ChAs values calculated on this randomized network are higher than those seen in our original dataset. This analysis showed our ChAs values to be substantially higher for 56 features, including all the ones that have high ChAs in the original dataset (**Figure S4B, Additional File 5**). It must be noticed that this type of procedure preserves the possible chromosomal correlation between features, as distances spanned by interactions are not substantially altered, but it completely rewires the interaction network making it very unlikely to see any promoter-promoter interaction. These two randomization approaches both interfere with the relationship between the topology of the interactions and the distance spanned by interactions.

To further test how distances spanned by our interactions affect ChAs, we established four representative threshold distances (10 Kb,100 Kb, 1000 Kb and 5000 Kb) for which we investigate to what extent the ChAs values reflect the correlations in feature values already found along the chromosome. We performed a targeted edge removal and show the

correlation of the ChAs measured at these four different distance thresholds compared to the ChAs measured on the full network. (**Figure S5**).

Even when removing all short-distance interactions from the PCHi-C network, we observe very similar values of ChAs (Pearson's r>0.82, p-value=0). We noticed that PcG features tend to acquire higher assortativity as the short distance connections are removed and, interestingly, the elongating form of RNAPII (S2p) tends to preserve ChAs at larger scale distances better than the other RNAPII variants (**Figure S5D**).

Figure S2: ChAs robustness to edge and node removal

ChAS values measured as the network is reduced by different approaches. A) Random edge removal, B) Targeted removal of nodes with highest EZH2 abundance. EZH2 ChAs shown in pink, ChAs of other PcG features shown in black and other features in grey.

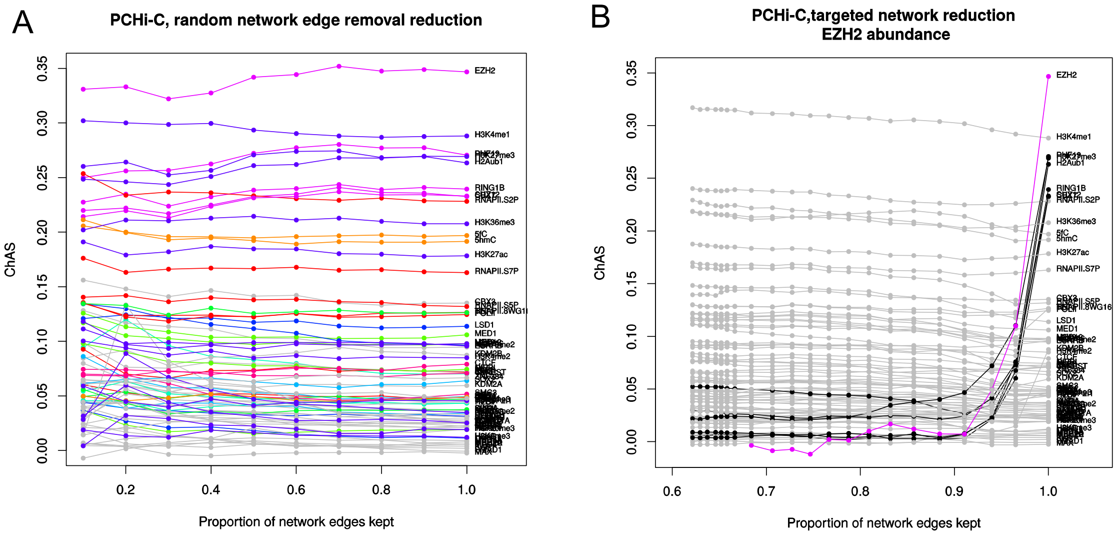

Figure S3: Schematic description of randomization procedures

A comparison of the two randomization procedures used focusing on neighborhood of interaction of one specific promoter (labelled with *). Panel I shows the real network (promoter nodes shown with thicker outline) and how we mapped feature values on 200bp chromosome segments (rectangles) to fragments (ovals). Here feature value is represented as red when present and white when absent to simplify. Panel II shows the first randomization strategy, where we permute the labels of each fragment but preserve the network wiring. Panel III shows a randomization in which the feature distribution along the genome is preserved but different fragments (at distances comparable to the original ones) are made to interact with the bait promoter fragment.

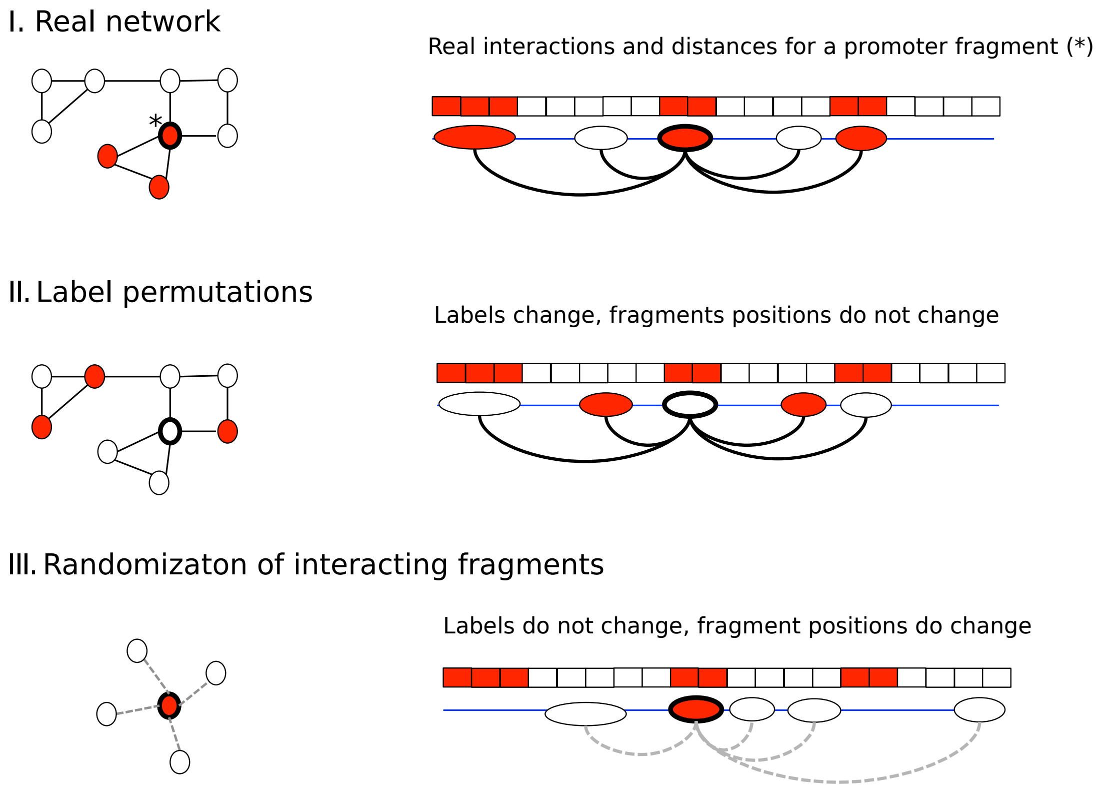

Figure S4: Results of randomization procedures

A) Network preserving feature permutation randomization (See Figure S3 II); B) Randomization of interactions preserving feature chromosomal correlation (See Figure S3 III).

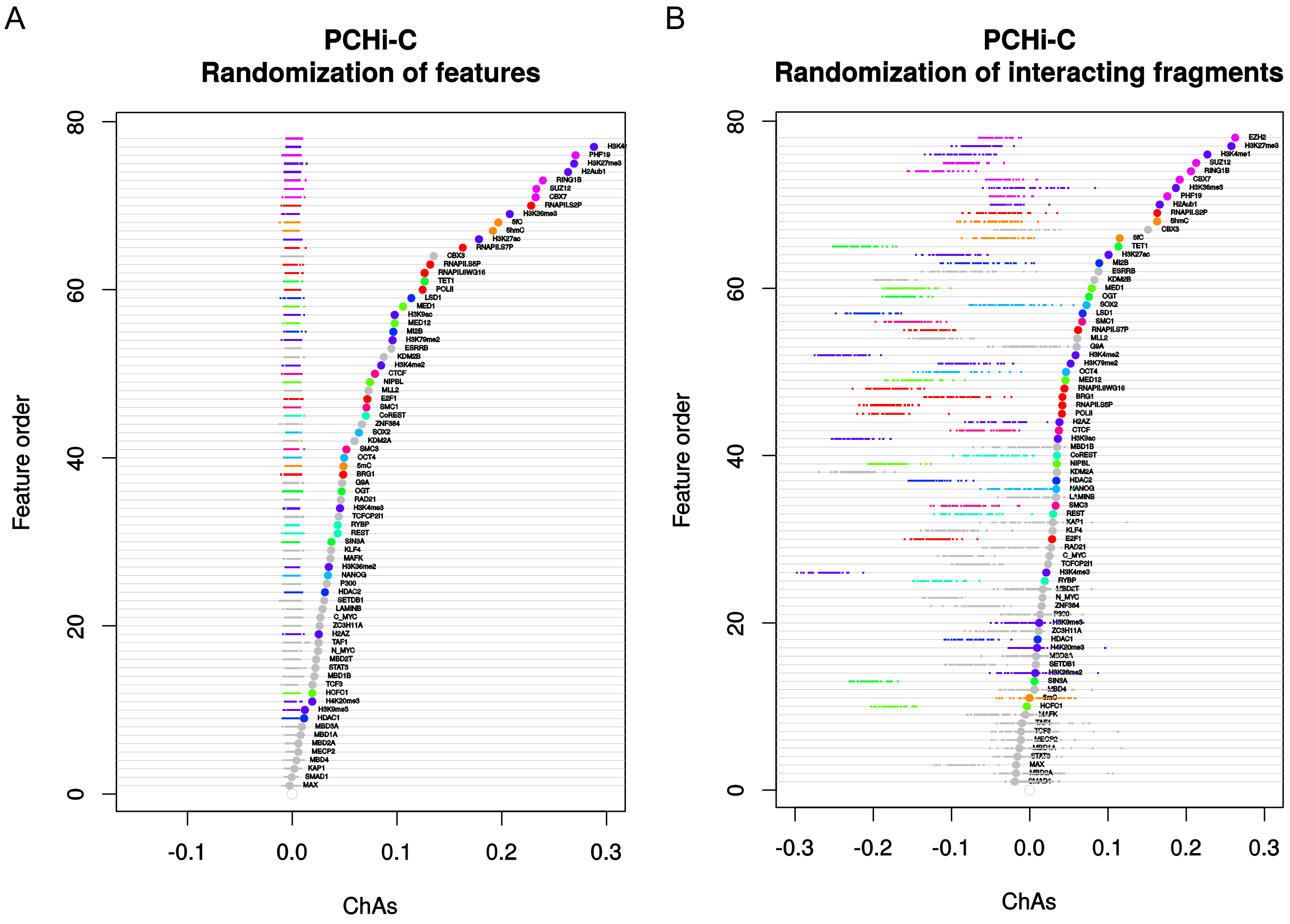

Figure S5: ChAs correlation of network after distance filtering

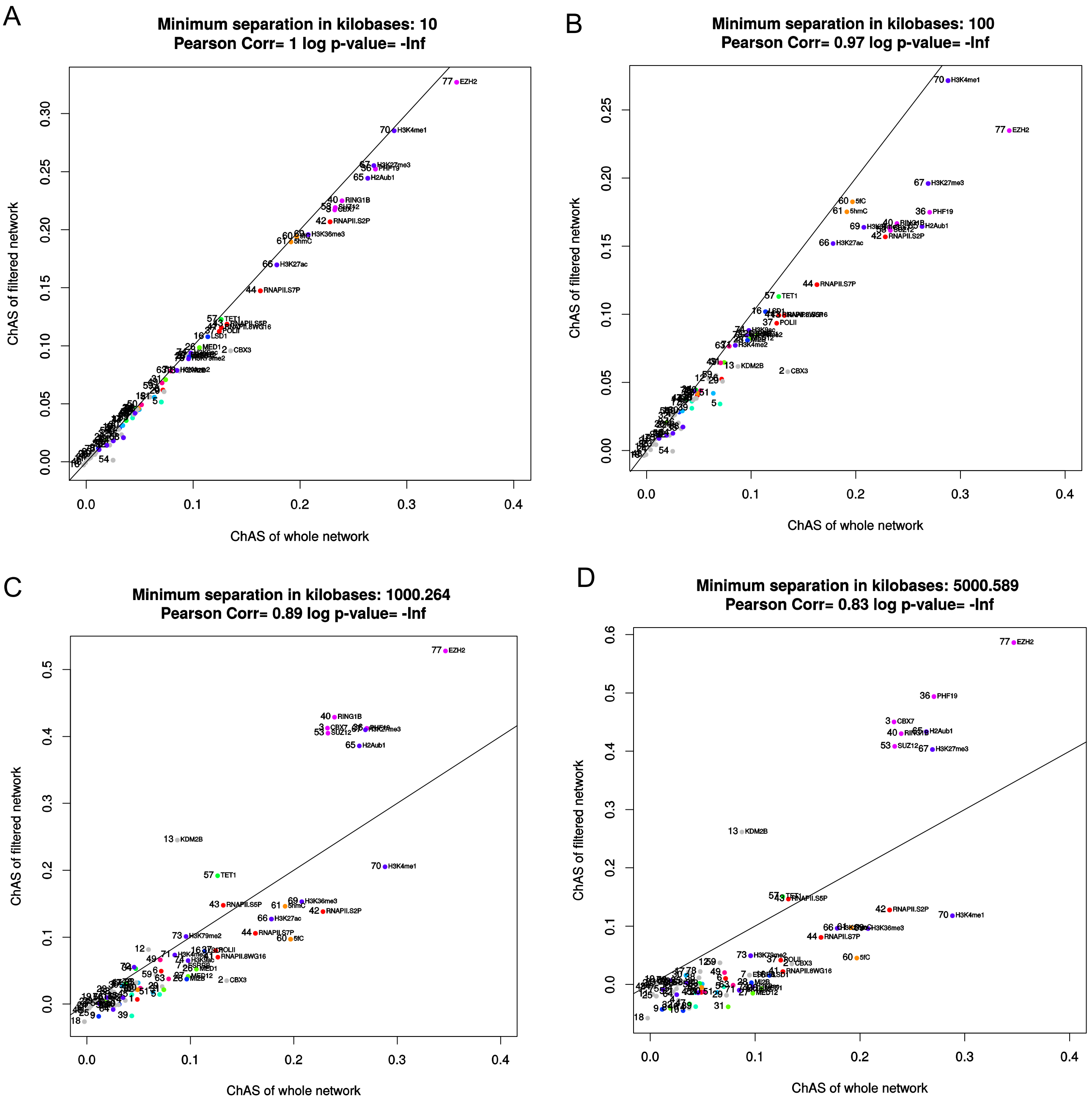

Text S2 Comparison of ChAs with traditional approaches

To further investigate the meaning of the ChAs, we performed extensive analysis of the relationship between PCHiC network measures and the presence of different epigenomic features. For different network node properties we identified the 500 nodes with the highest value and then measured the abundance of each feature in these nodes compared to the abundance in the whole network, considering this as a measure of association between the feature and the node property.

We then compared ChAs versus these enrichment values (**Figure S6**). For example, we might expect specific features to be present mostly on highly connected nodes, and indeed we find the abundance of KDM2B as well as PcG features to be higher in hubs (nodes with high degree). However, not all these features are characterised by high ChAs and the correlation is moderate (Pearson's $r = 0.49$, **Figure S6A**).

We repeated this enrichment analysis using the top 500 nodes with highest transitivity (also called clustering coefficient), which represents the extent to which neighbours of a node are connected with each other (**Figure S6B**). PcG features appear to be under-enriched in nodes with high transitivity, whereas Methyl Binding Domain proteins seem to be more highly abundant in nodes with high transitivity.

Figure S6: Comparison of ChAs with enrichment of features in important nodes.

A) ChAs vs. enrichment in top 500 nodes for degree. B) ChAs vs. enrichment in top 500 nodes for transitivity.

A

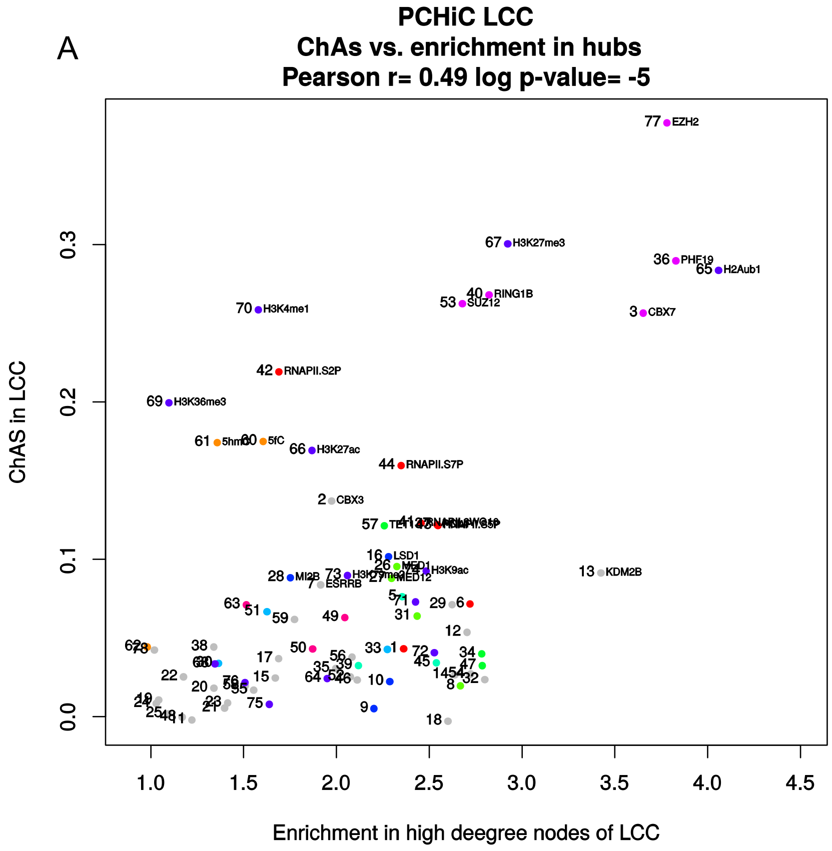

B

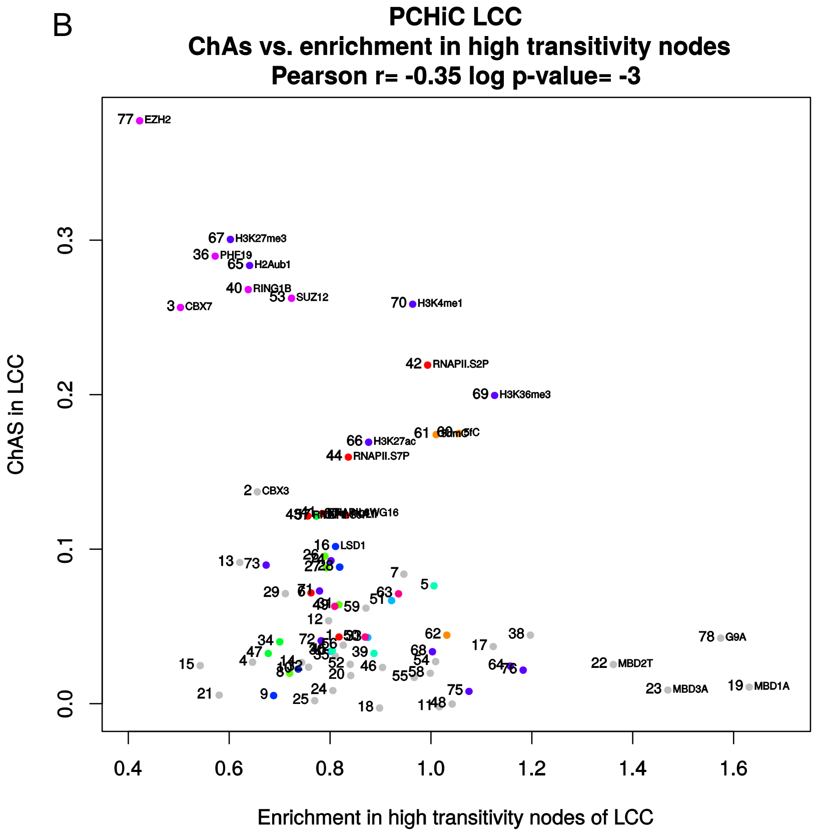

Text S3: Network community finding

We performed an enrichment analysis for each epigenomic feature to see whether features with high ChAs also showed local enrichment in specific chromatin communities. We proceeded to define network modules or communities (**Figure S7A**).

First we defined network communities using a fast greedy algorithm (as implemented in igraph with *fastgreedy.community()* function) and identified 147 dfferent ones. Then we ran the ModuLand overlapping community detection algorithm (as implemented in the ModuLand package in Cytoscape (Shannon et al., 2003; Szalay-Beko et al., 2012)), which identified 979 different overlapping modules. For both cases we then proceeded to estimate enrichment for the different features in these communities. For each of the two methods we did the following: we first calculated the ratio of abundance in nodes within one community and the total abundance in the network. Then we performed 100 permutations of assignment of nodes to communities, therefore preserving the communities' sizes and recalculated the ratio of abundance in the new communities over the total network. We used the number of times that randomized communities would have higher enrichment for the feature than the original community as an estimate of significance of the enrichment.

Finally we compared the number of communities that were enriched for each feature between the two algorithms. The two algorithms gave quite comparable results, despite the big difference in number of communities detected (therefore module size) and the fact that ModuLand communities are defined as having the possibility of overlapping (a node assigned to more than one community). **Figure S7B** shows the number of modules enriched for each of the features in the two approaches and it can be seen that high ChAs features (such as PcG and RNAPII) were found to be enriched in a moderate number of modules, contrary to more abundant features such as H3K4me1 and 5hmC. This shows the difference between the global character of assortativity measurements compared to local enrichment approaches.

Figure S7: Enrichment of features in chromatin communities

A) Schema of clustering vs assortativity calculation, highlighting the global versus local characters of the two approaches. Bottom: Comparison of proportion of modules enriched using all epigenomic features using the ModuLand and the Fast Greedy approach. B) Comparison of proportion of communities found with each algorithm to be enriched in all the features.

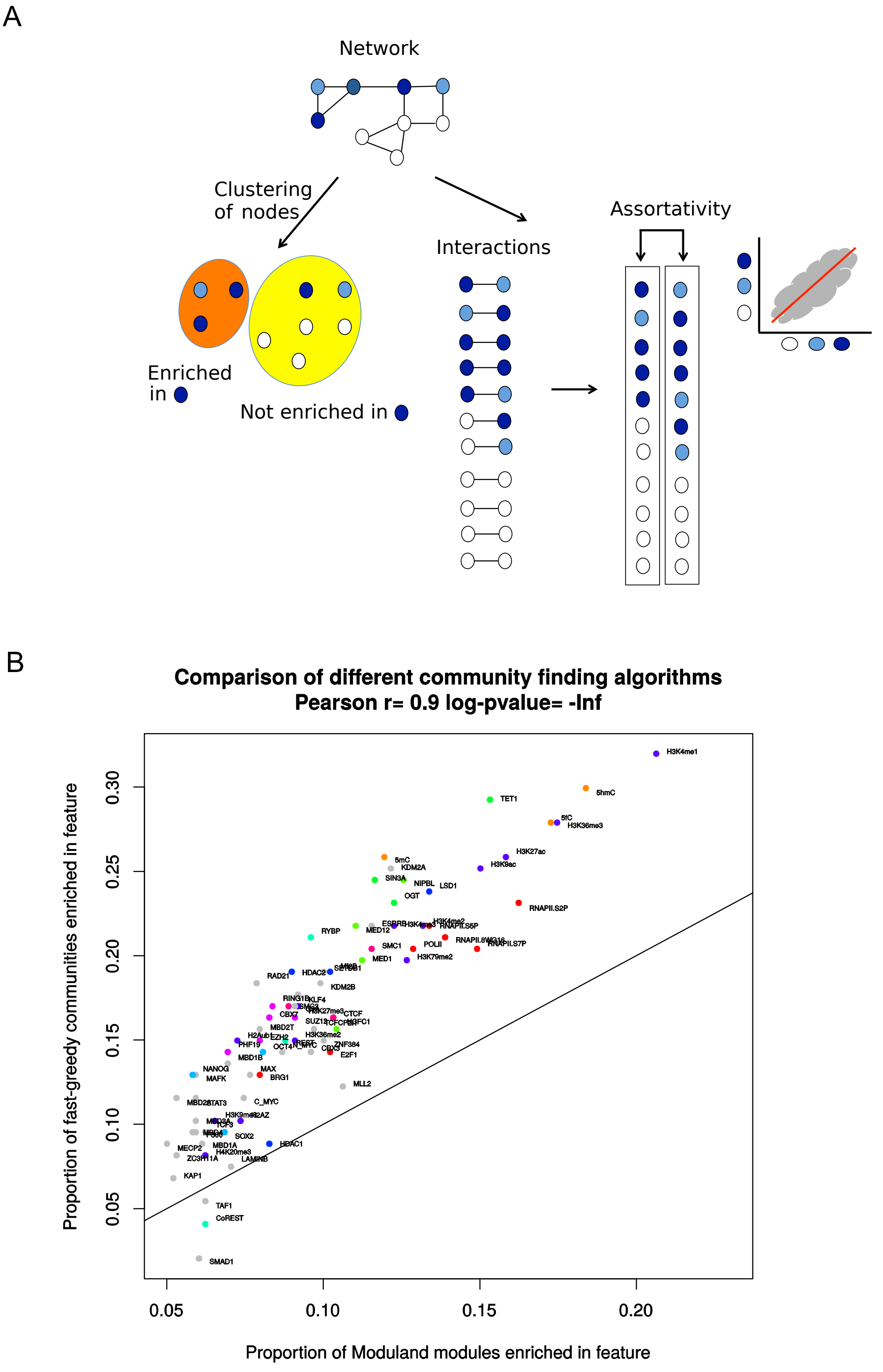

Figure S8: HiCap chromatin interaction network

A) HiCap network, Promoter nodes and P-P constacts are shown in red, while Other-ends and P-O contacts are shown in blue. B) HiCap network ChAs vs. abundance. C) Comparison of ChAs between HiCap and PCHiC.

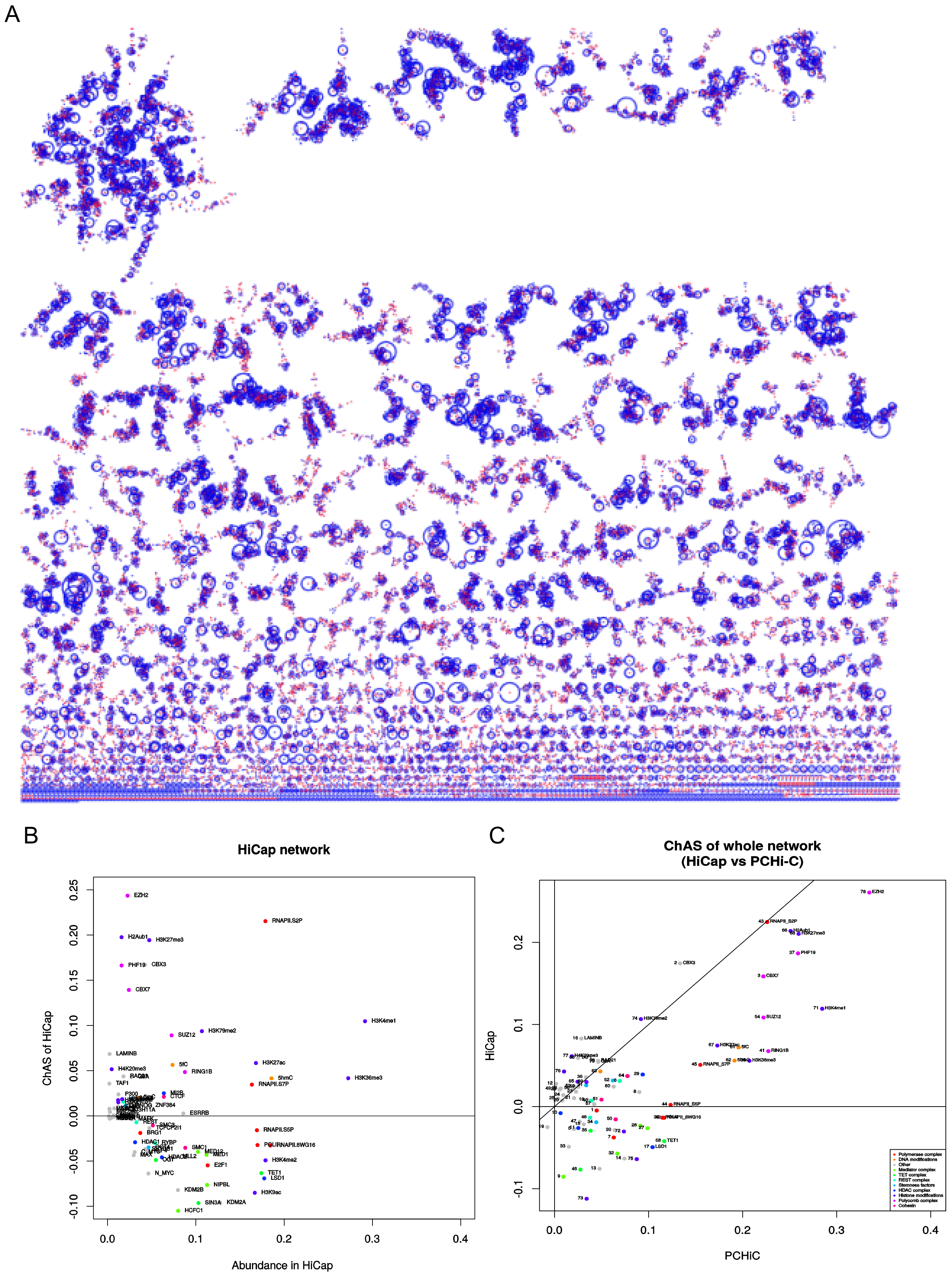

Figure S9: ChIA-PET networks, SMC1 and RNAPII and network statistics

ChAs Versus abundance in ChIA-PET networks. A) SMC1 ChIA-PET ChAs vs. abundance, B) RNAPII ChIA-PET ChAs vs. abundance. C) SMC1 ChIA-PET network visualized using Cytoscape. D) RNAPII ChIA-PET network visualized using Cytoscape. E) Comparison of different networks measures in all studied networks.

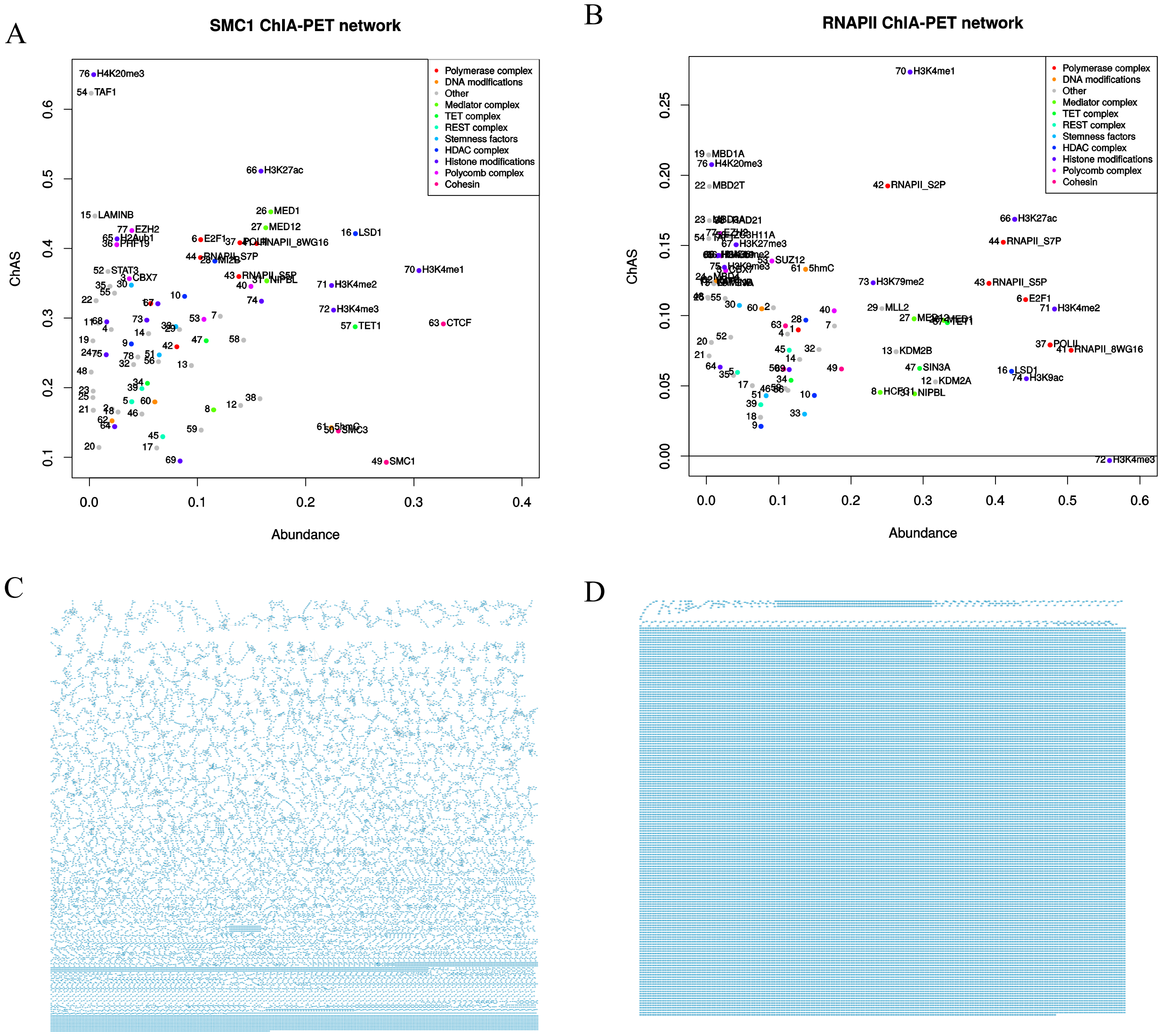

E

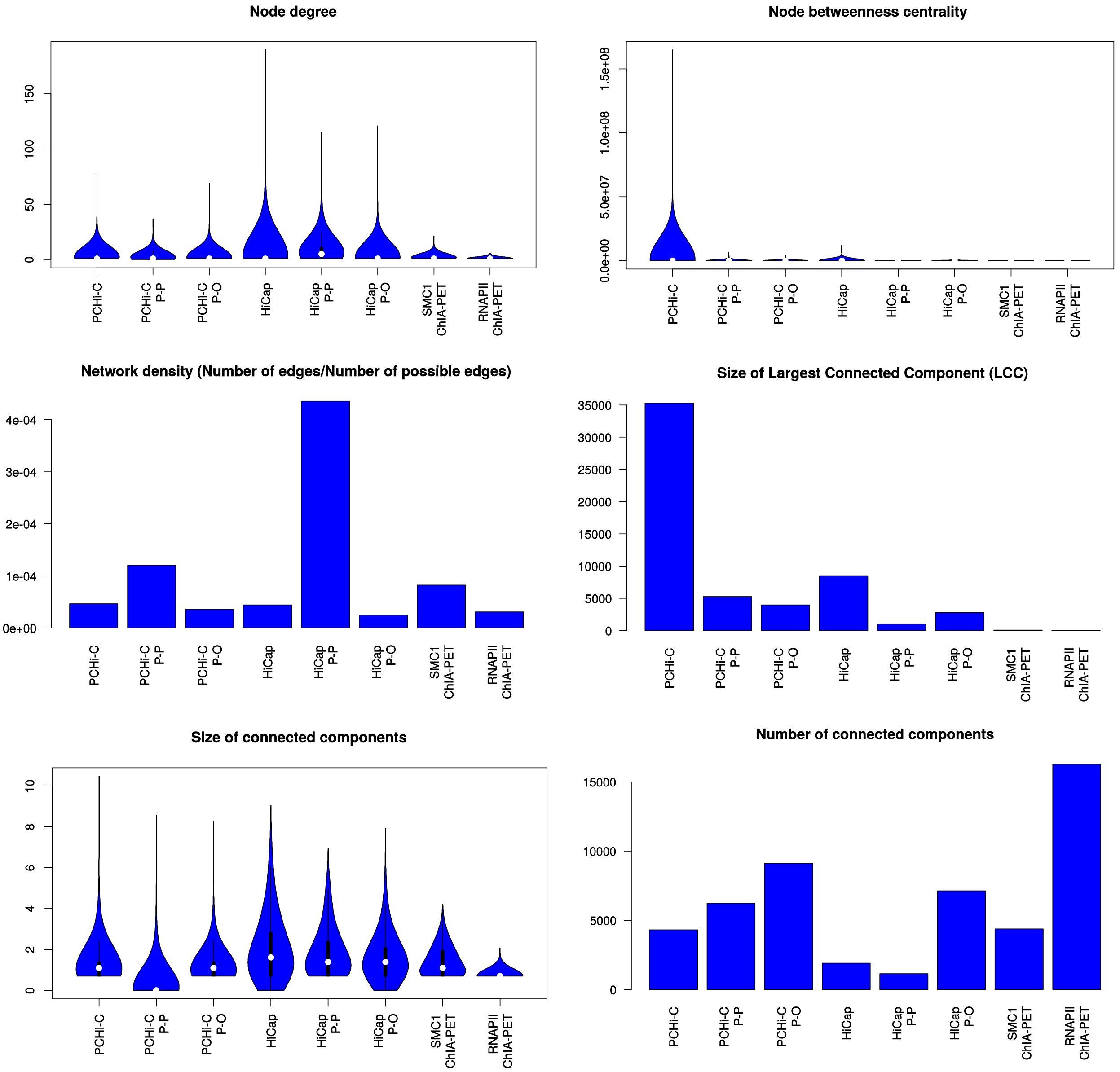
Node degree
0
50
100
150
PCHi-C
PCHi-C P-P
PCHi-C P-O
HiCap
HiCap P-P
HiCap P-O
SMC1 ChIA-PET
RNAPII ChIA-PET
Node betweenness centrality
0.0e+00
5.0e+07
1.0e+08
1.5e+08
Network density (Number of edges/Number of possible edges)
0e+00
1e-04
2e-04
3e-04
4e-04
Size of Largest Connected Component (LCC)
0
5000
10000
15000
20000
25000
30000
35000
Size of connected components
0
2
4
6
8
10
Number of connected components
0
5000
10000
15000

Figure S10: Comparison of ChAs in 3 networks against PCHiC

A) HiCap vs. PCHiC; B) SMC1 ChIA-PET vs PCHiC; C) RNAPII ChIA-PET vs PCHiC.

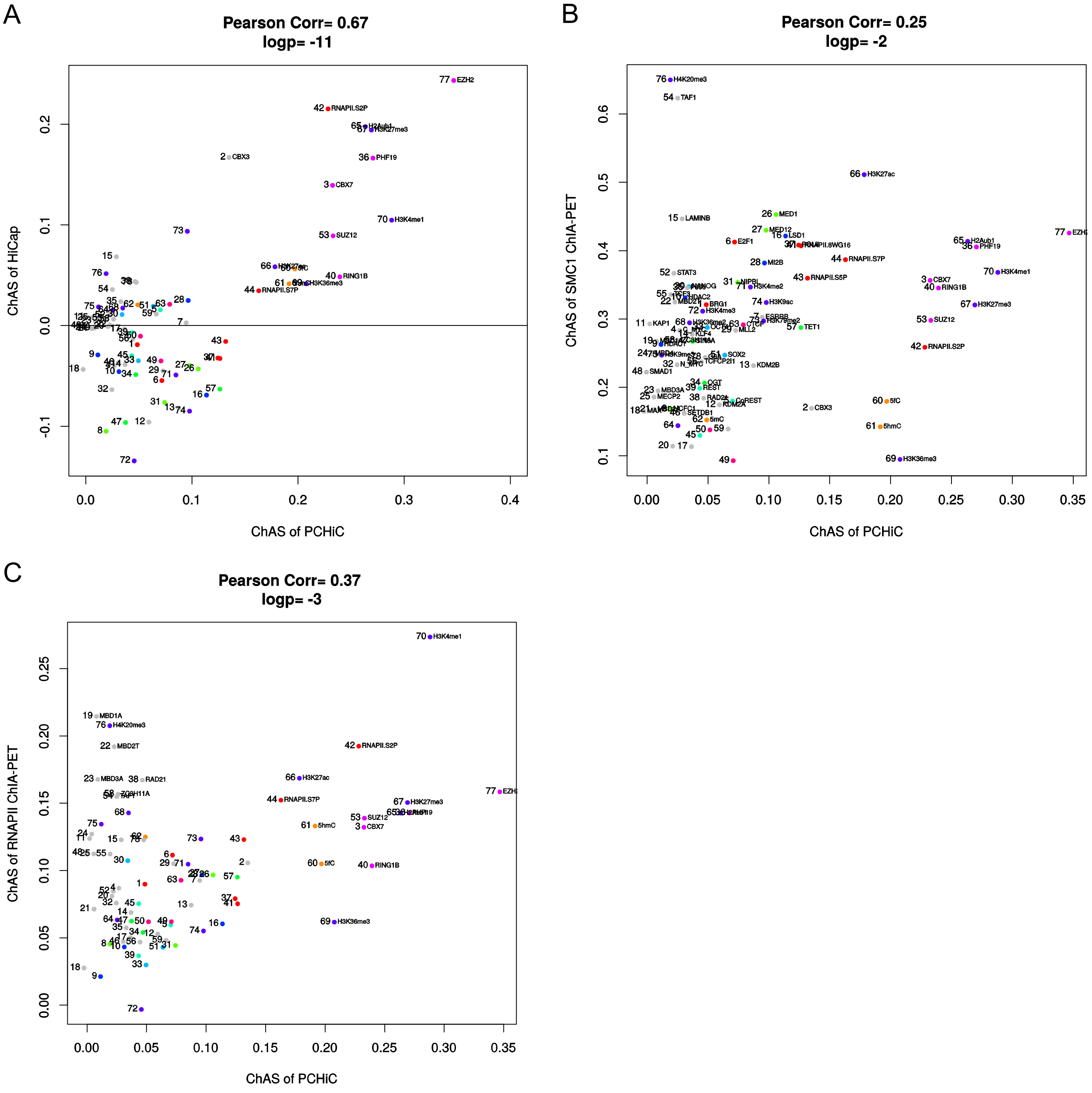

Figure S11: Comparison of ChAs in 3 networks against HiCap and RNAPII ChIA-PET vs SMC1 ChIA-PET

A) SMC1 ChIA-PET vs HiCap; B) RNAPII ChIA-PET vs HiCap; C) RNAPII ChIA-PET vs HiCap.

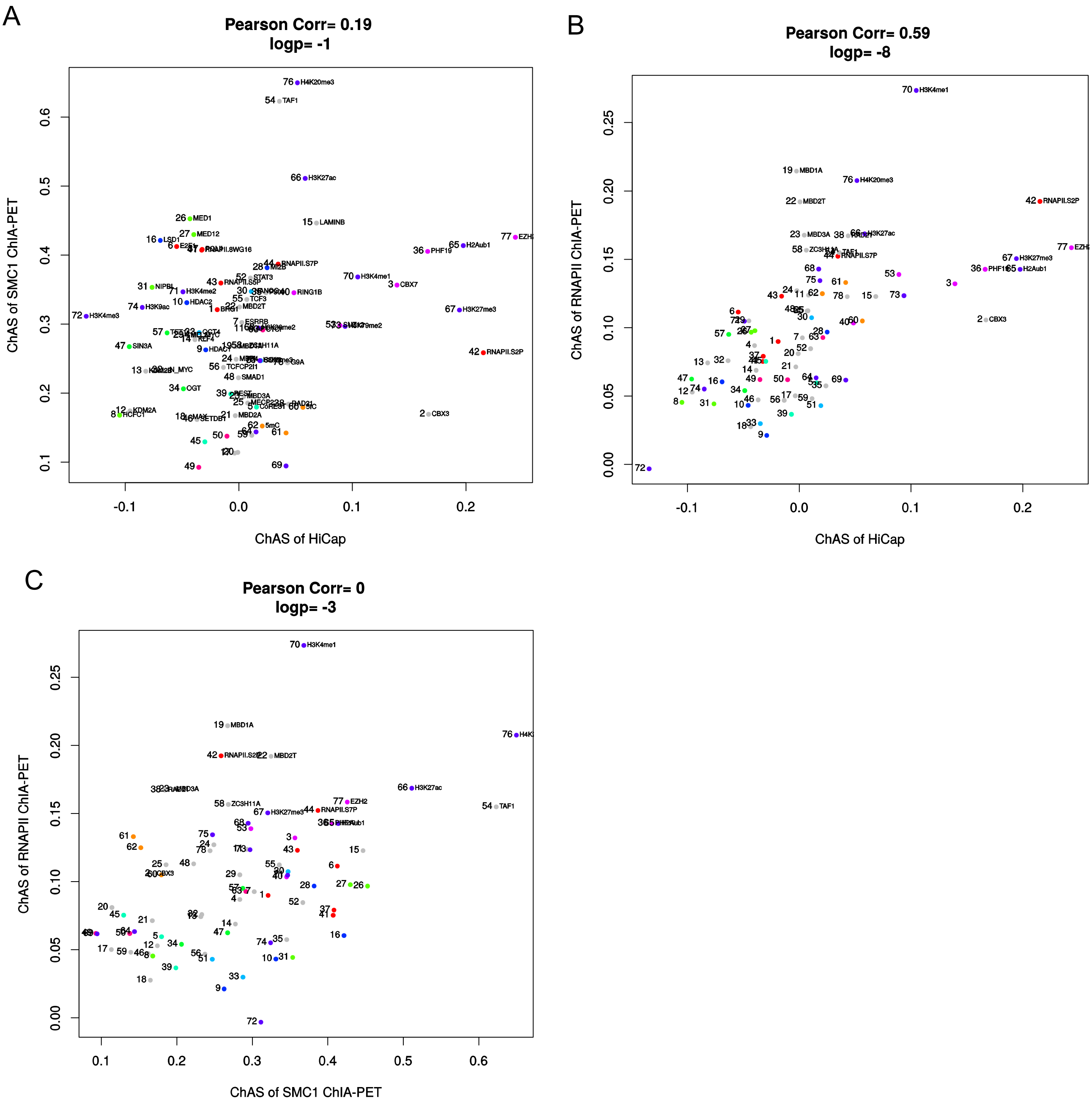

Figure S12: Comparison of ChAs P-P vs. P-O

A) PCHi-C P-P subnetwork ChAs vs. abundance, B) PCHi-C P-O subnetwork ChAs vs. abundance, C) Hi-Cap P-P subnetwork ChAs vs. abundance, D) Hi-Cap P-O subnetwork ChAs vs. abundance, E) ChAs P-P subnetwork, HiCap vs. PCHi-C, F) ChAs P-O subnetwork, HiCap vs. PCHi-C, G) ChAs P-O subnetwork vs. ChAs P-P subnetwork in HiCap, H) ChAs P-O subnetworks vs. ChAs P-P subnetworks from 100 rewired PCHi-C networks. The wirings preserved the number of P-P and P-O interactions (see Methods for details).

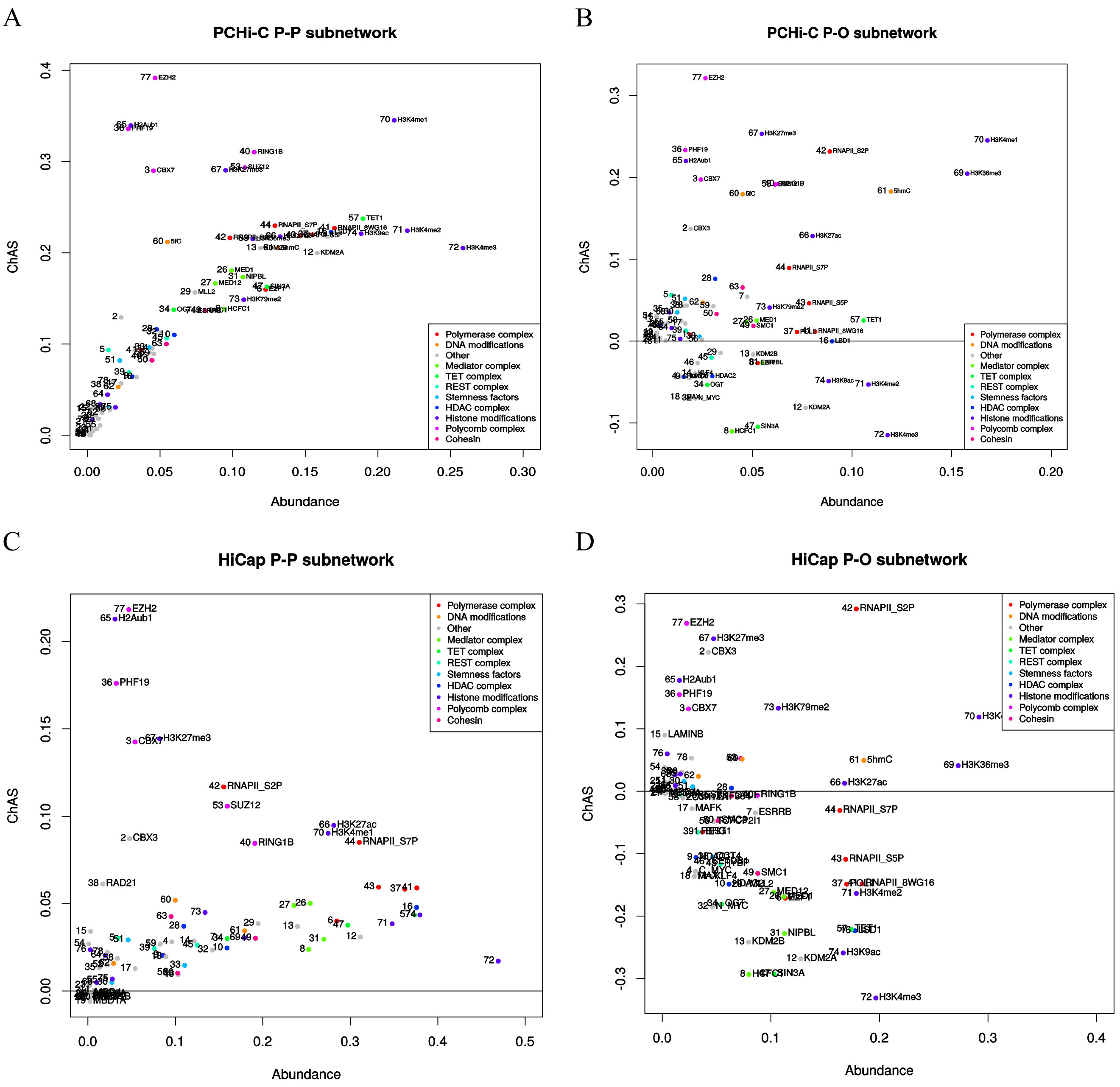

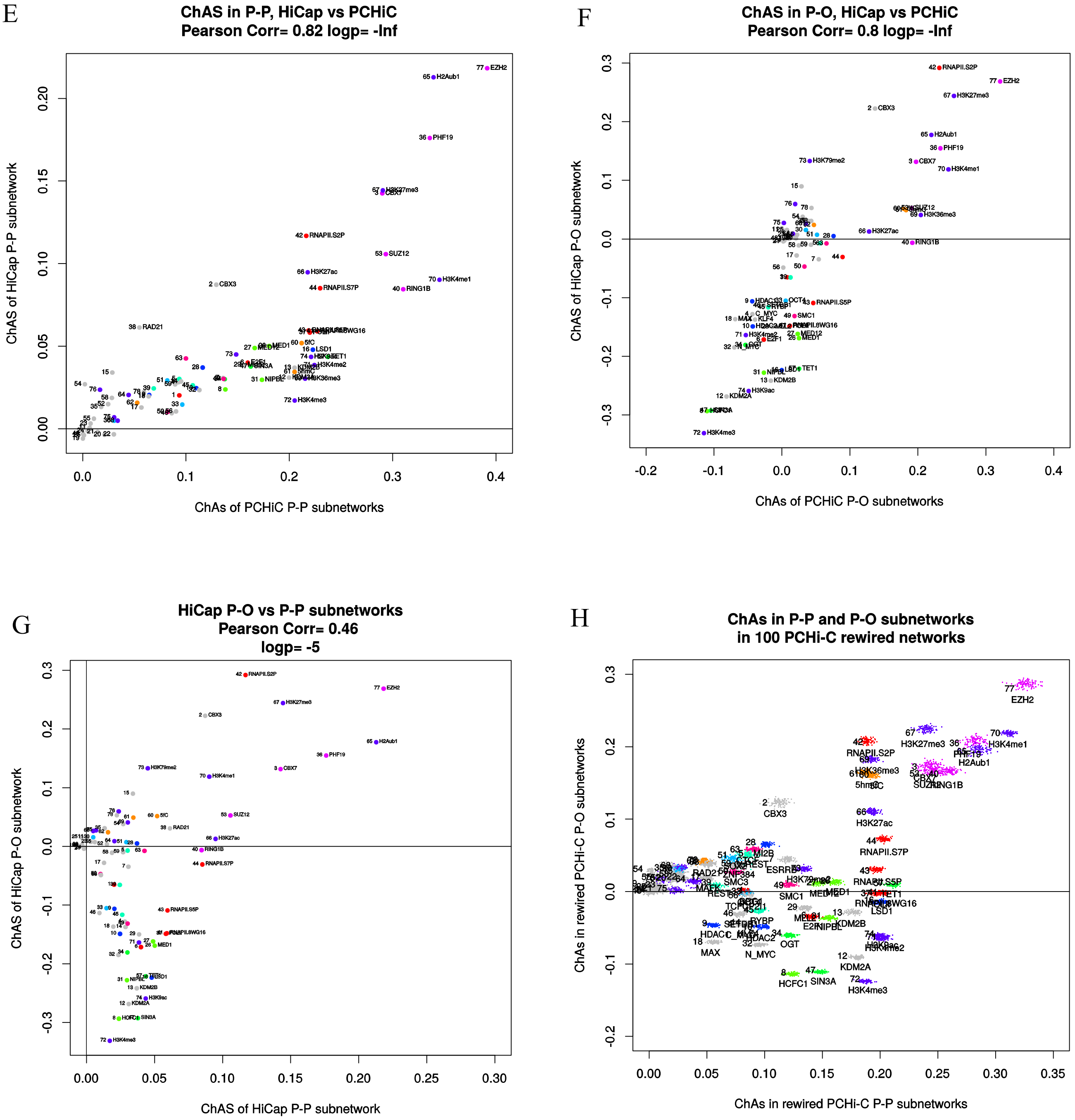
E
ChAS in P-P, HiCap vs PCHiC
Pearson Corr= 0.82 logp= -Inf
ChAS of HiCap P-P subnetwork
ChAs of PCHiC P-P subnetworks
F
ChAS in P-O, HiCap vs PCHiC
Pearson Corr= 0.8 logp= -Inf
ChAS of HiCap P-O subnetwork
ChAs of PCHiC P-O subnetworks
G
HiCap P-O vs P-P subnetworks
Pearson Corr= 0.46
logp= -5
ChAS of HiCap P-O subnetwork
ChAS of HiCap P-P subnetwork
H
ChAs in P-P and P-O subnetworks
in 100 PCHi-C rewired networks
ChAs in rewired PCHi-C P-O subnetworks
ChAs in rewired PCHi-C P-P subnetworks

Figure S13 Core and periphery

ChAs value calculated only on LCC of PCHi-C networks vs. ChAs calculated only on other connected components of the PCHi-C network.

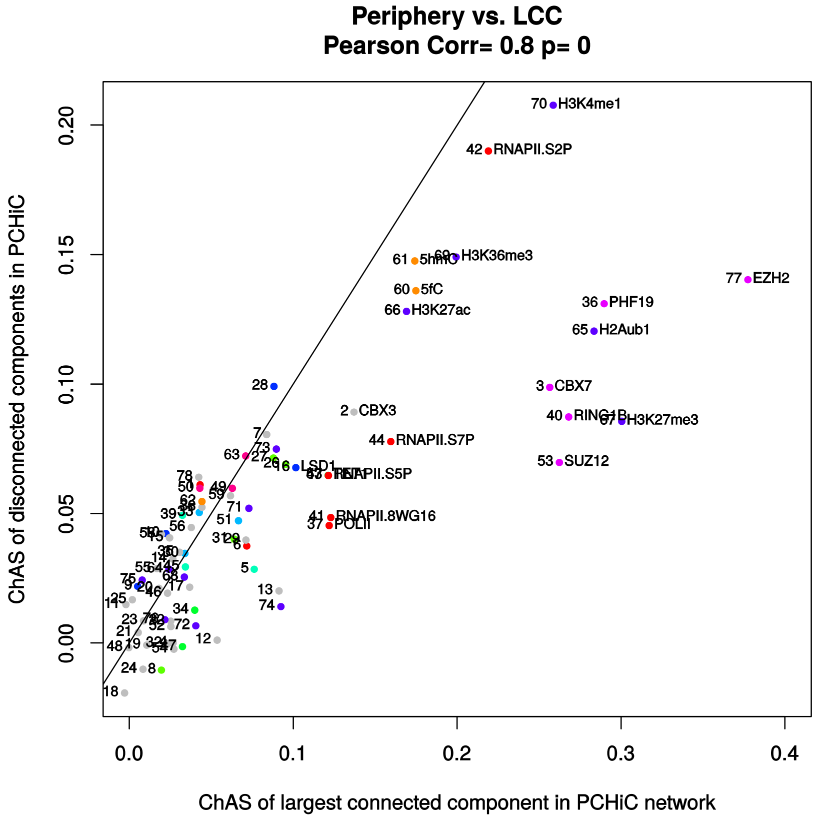

Text S4: Party and date hubs

The concept of Date and Party hubs was first introduced by (Han et al., 2004) to distinguish two different types of hubs, highly connected nodes in in protein interaction networks. In those networks it was expected that some proteins would interact with many others at the same time, as in a stable complex, while others would have different interactions at different moments. Thus interactions accompanied by higher expession correlation of the interacting proteins could identify party hubs. In other networks it is harder to determine which nodes have a date or party character. However, using ModuLand and measuring the bridgeness of nodes, that is the number of overlapping modules that each node belongs to, one can estimate date/party hub properties of nodes. More specifically, as suggested in (Kovács, Palotai, Szalay, & Csermely, 2010) date hubs would be nodes that show more prominent bridgeness than betweenness centrality and party hubs would instead be more central and have less bridgeness.

We identified the top 500 nodes with highest betweenness and the top 500 nodes with higher bridgeness and then looked at relative abundance of features in these nodes compared to the whole network. We then interpreted features that are abundant in high bridgeness but not in high betweenness nodes to be equivalent to date features. On the other side features that are more abundant in high betweenness and not high bridgeness nodes can be considered to be more party-hub features. We find PcG features to have a clear party-hub profile whereas no feature has a clear date-hub profile (**Figure S14**).

Figure S14 Party/date hubs

A) ChAs vs enrichment of features in top 500 betweenness centrality nodes, B) Enrichment of features in top 500 bridgeness nodes vs enrichment of features in top 500 betweenness centrality nodes (see Figure 4B in the main text for ChAs vs enrichment in top bridgness nodes).

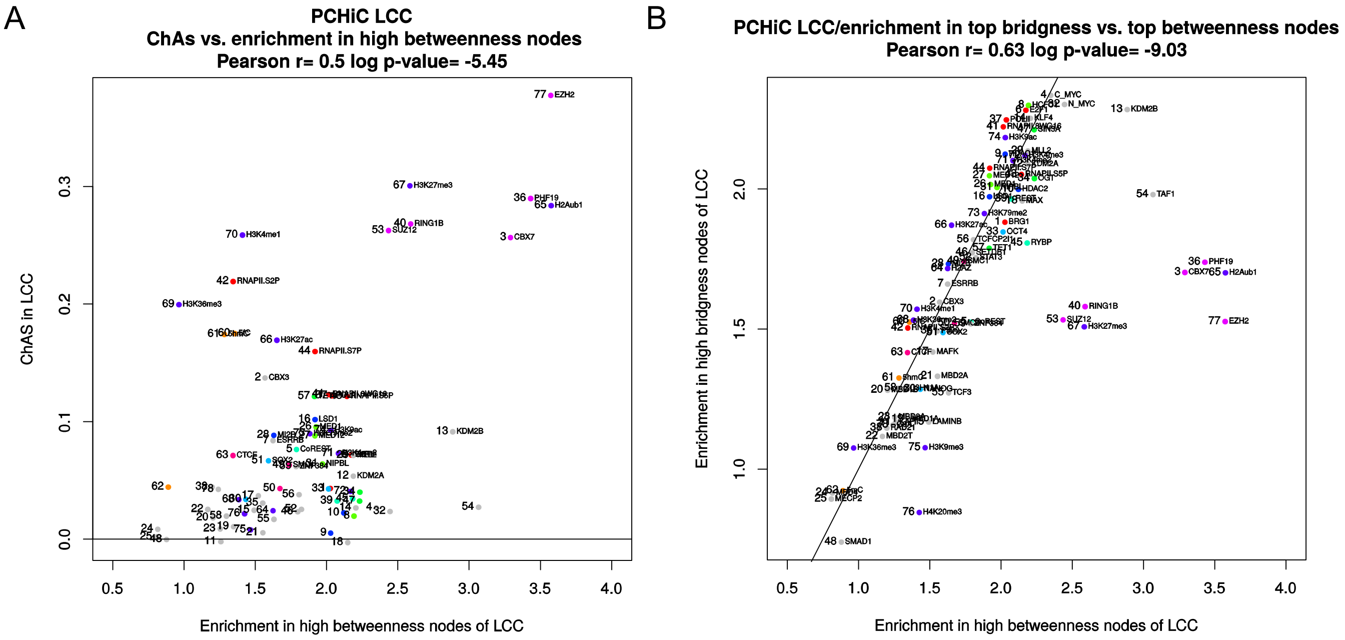

S15 Heatmap of correlations between enrichment of features in different chromatin communities.

Heatmap of the correlation of enrichment of features in 979 different chromatin communities identified with ModuLand, for all features with ChAs >0.1 in the LCC. Four clusters can be easily identified, which are the same as in Figure 4: I PcG features , II TET1 related features, III Non-elongating RNAPII, IV Elongating RNAPII.

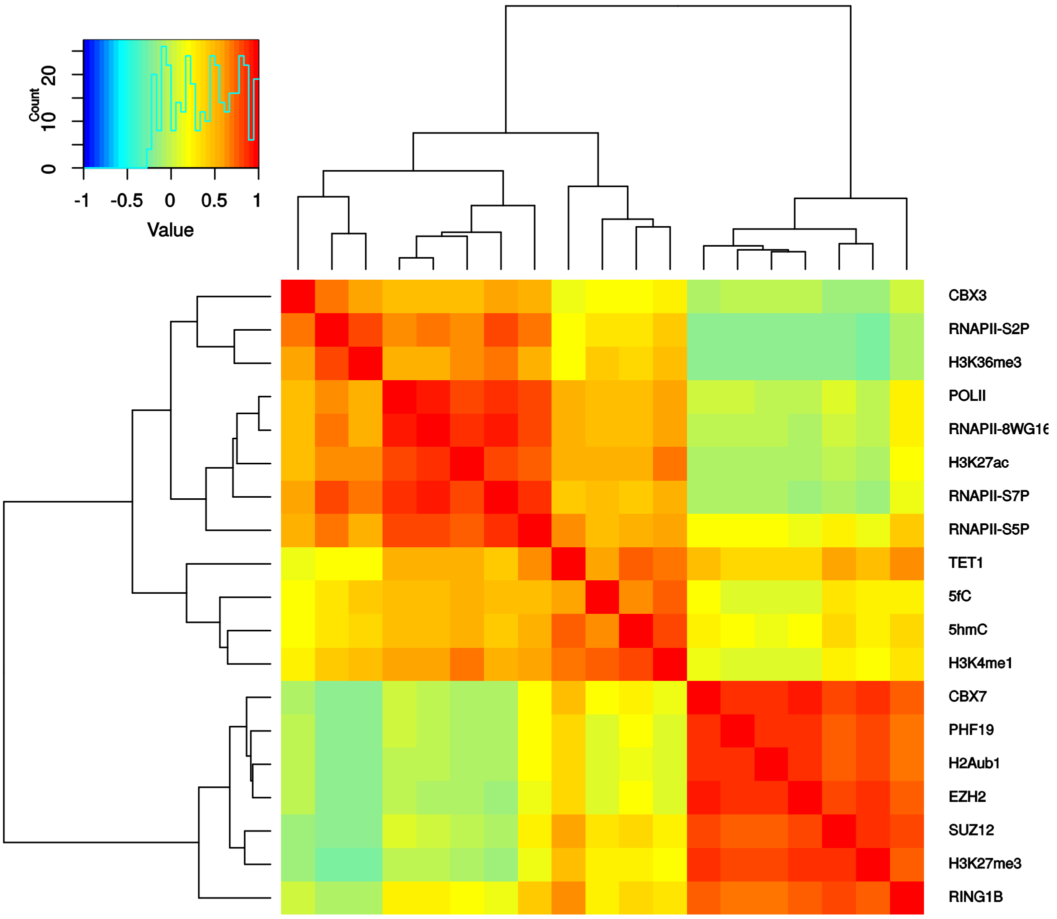

Figure S16: ChAs and abundance for different RNAPII variants in P-P and P-O subnetworks and their rewirings

A) Comparison of ChAs for 100 rewired PCHi-C, HiCap, RNAPII ChIA-PET and SMC1 ChIA-PET networks; B) ChAS in 100 PCHi-C rewired networks, comparison of P-P and P-O for different RNAPII variants; C) Abundance in PCHi-C; D) ChAS in 100 Hi-Cap rewired networks, comparison of P-P and P-O for different RNAPII variants; E) Abundance in HiCap; F) Comparison of ChAs in contacts between promoters and different types of other ends for 100 rewired PCHi-C and HiCap networks.

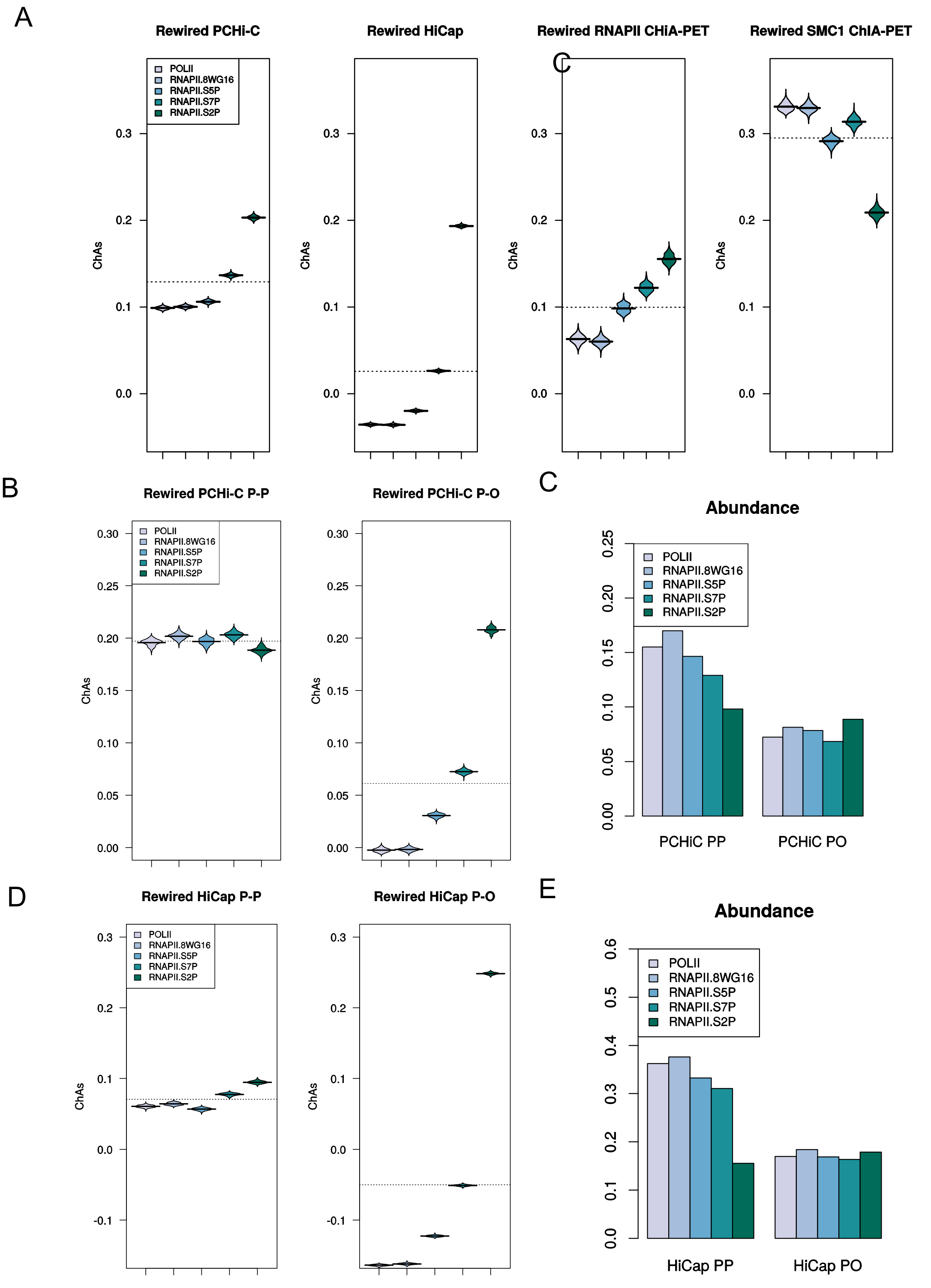

F

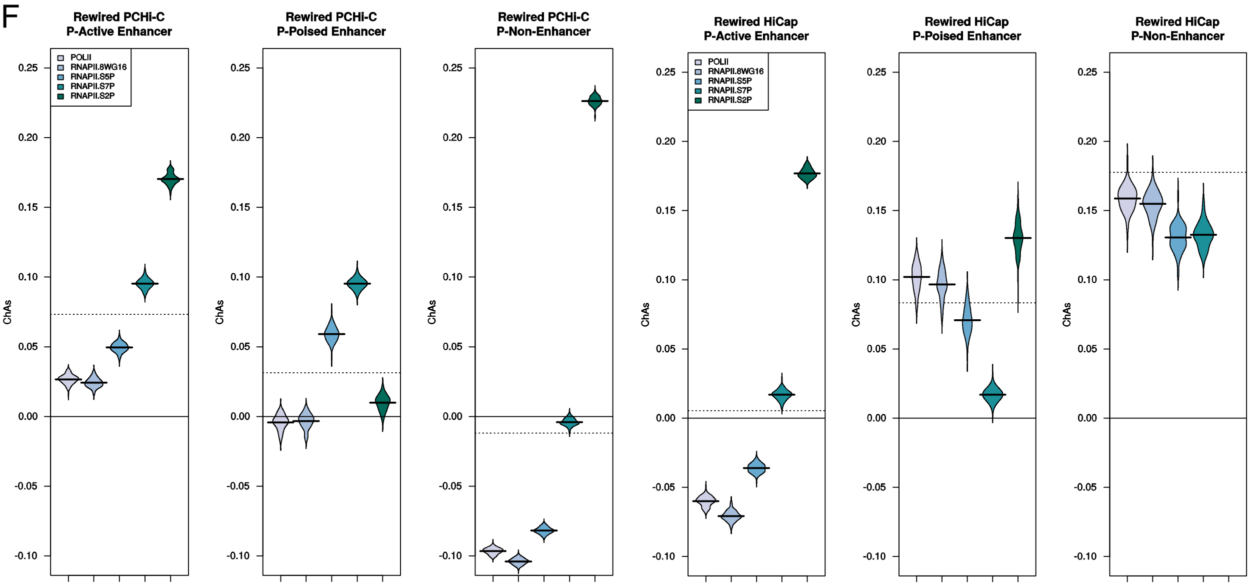
Rewired PCHi-C
P-Active Enhancer
Rewired PCHi-C
P-Poised Enhancer
Rewired PCHi-C
P-Non-Enhancer
Rewired HiCap
P-Active Enhancer
Rewired HiCap
P-Poised Enhancer
Rewired HiCap
P-Non-Enhancer
POLII
RNAPII.8WG16
RNAPII.S5P
RNAPII.S7P
RNAPII.S2P
ChAs
0.25
0.20
0.15
0.10
0.05
0.00
-0.05
-0.10

Figure S17: Abundance of features in Other end fragments with no H3K4me1 enhancer mark

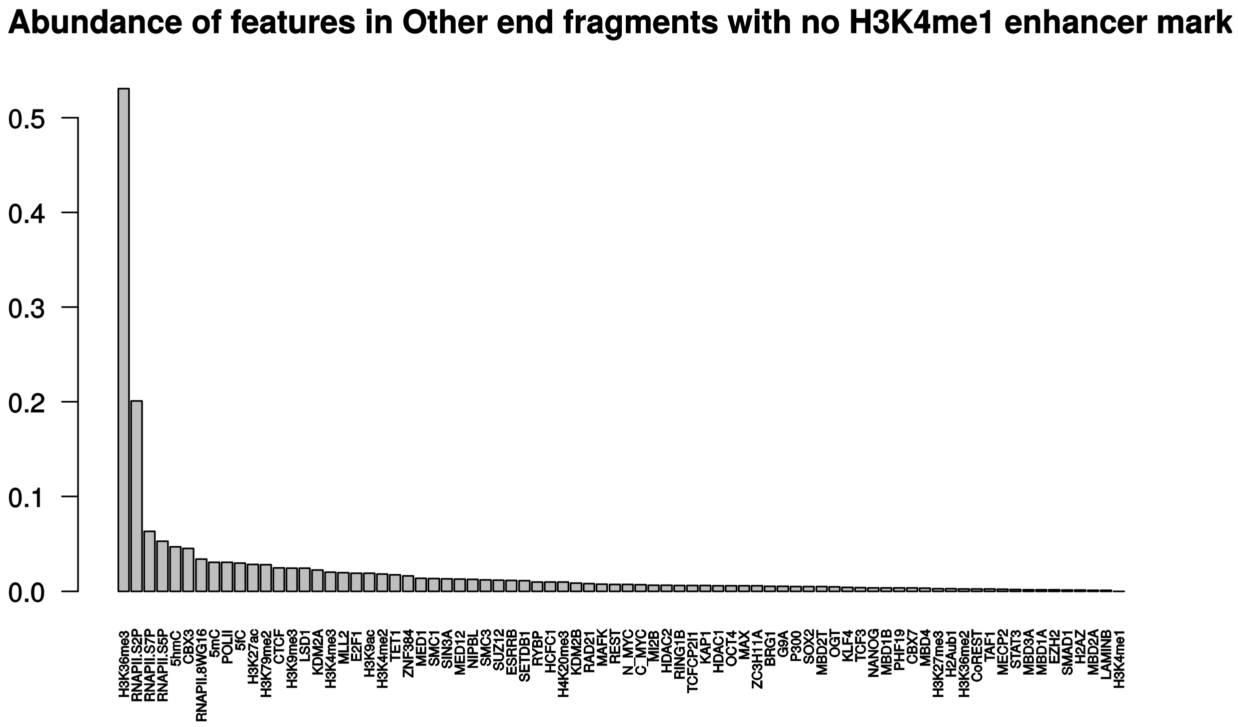

Figure S18: Comparison of presence of different RNAPII variants in P and O fragments

A) PCHi-C P fragments; B) PCHi-C O fragments; C) HiCap P fragments; B) HiCap O fragments;

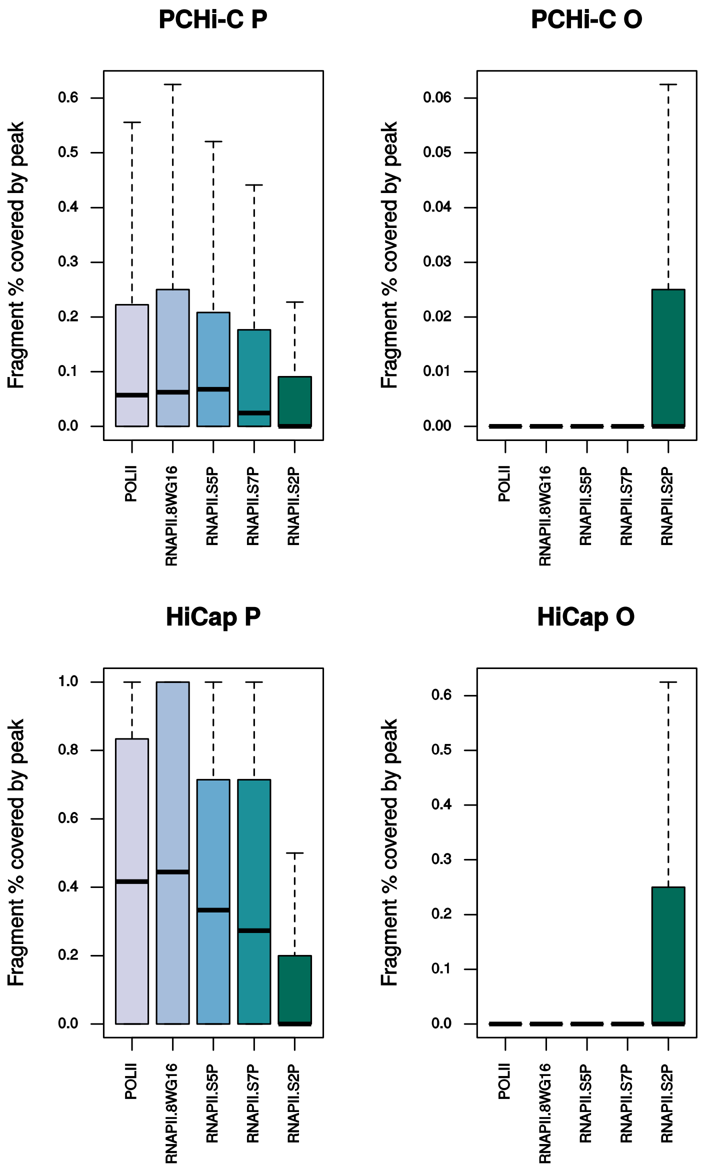

Table S1: Overlap of fragments in different chromatin interaction networks

Proportion of overlap over total in % in the brackets.

	SMC1	RNAPII	PCHi-C P	PCHi-C O	HiCap P	HiCap O
SMC1						
RNAPII	4748(20)					
PCHi-C P	4720(20)	19150(58)				
PCHi-C O	6031(25)	6436(19)				
HiCap P	2261(9)	16022(48)	8131(43)	674(2)		
HiCap O	3799(16)	8009(24)	5594(30)	7464(21)		
Total	24052	33126	18906	3644	15897	72025

References

Han, J.-D. J., Bertin, N., Hao, T., Goldberg, D. S., Berriz, G. F., Zhang, L. V, … Vidal, M. (2004). Evidence for dynamically organized modularity in the yeast protein-protein interaction network. *Nature*, *430*(6995), 88–93. doi:10.1038/nature02555

Kovács, I. A., Palotai, R., Szalay, M. S., & Csermely, P. (2010). Community landscapes: an integrative approach to determine overlapping network module hierarchy, identify key nodes and predict network dynamics. *PLoS ONE*, *5*(9), 14. doi:10.1371/journal.pone.0012528

Sahlén, P., Abdullayev, I., Ramsköld, D., Matskova, L., Rilakovic, N., Lötstedt, B., … Sandberg, R. (2015). Genome-wide mapping of promoter-anchored interactions with close to single-enhancer resolution. *Genome Biology*, *16*(1), 156. doi:10.1186/s13059-015-0727-9

Shannon, P., Markiel, A., Ozier, O., Baliga, N. S., Wang, J. T., Ramage, D., … Ideker, T. (2003). Cytoscape: a software environment for integrated models of biomolecular interaction networks. *Genome Research*, *13*(11), 2498–504. doi:10.1101/gr.1239303

Szalay-Beko, M., Palotai, R., Szappanos, B., Kovács, I. A., Papp, B., & Csermely, P. (2012). ModuLand plug-in for Cytoscape: determination of hierarchical layers of overlapping network modules and community centrality. *Bioinformatics (Oxford, England)*, *28*(16), 2202–4. doi:10.1093/bioinformatics/bts352
